# Joslin on Cholera

**Published:** 1884-12

**Authors:** 


Joslin on Cholera.—A new edition of this valuable work on *Cholera* will be issued as our first supplement for 1885. It will be prepared by Dr. P. P. Wells.

